# COVID-19 HEART unveiling as atrial fibrillation: pathophysiology, management and future directions for research

**DOI:** 10.1186/s43044-023-00359-0

**Published:** 2023-04-30

**Authors:** Sri Harsha Kanuri, Prapthi Jayesh Sirrkay, Ayse Sena Ulucay

**Affiliations:** 1 Case Western Reserve University, Cleveland, OH 44106 USA; 2 University of Minnesota, Minneapolis, MN USA

**Keywords:** COVID-19, Atrial fibrillation, Arrhythmias, Atrial reentry, Atrial premature beats, Atrial substrate, Automaticity, Inflammation, Cytokine storm, Oxidative stress

## Abstract

**Background:**

COVID-19 infections are known to cause numerous systemic complications including cardiovascular disorders. In this regard, clinicians recently noticed that patients recovering from COVID-19 infections presented with diverse set of cardiovascular disorders in addition to those admitted to ICU (intensive care unit). COVID-19 heart has multifaceted presentation ranging from dysrhythmias, myocarditis, stroke, coronary artery disease, thromboembolism to heart failure. Atrial fibrillation is the most common cardiac arrhythmia among COVID-19 patients. In the background section, we briefly discussed epidemiology and spectrum of cardiac arrhythmias in COVID-19 patients.

**Main body:**

In this state-of-the-art review we present here, we present the information regarding COVID-19-induced A-fib in sections, namely mechanism of action, clinical presentation, diagnosis and treatment. Unfortunately, its occurrence significantly increases the mortality and morbidity with a potential risk of complications such as cardiac arrest and sudden death. We included separate sections on complications including thromboembolism and ventricular arrhythmias. Since its mechanism is currently a gray area, we included a separate section on basic science research studies that are warranted in the future to comprehend its underlying pathogenic mechanisms.

**Conclusions:**

Taken together, this review builds upon the current literature of COVID-19-induced A-fib, including pathophysiology, clinical presentation, treatment and complications. Furthermore, it provides recommendations for future research moving forward that can open avenues for developing novel remedies that can prevent as well as hasten clinical recovery of atrial fibrillation in COVID-19 patients.

**Supplementary Information:**

The online version contains supplementary material available at 10.1186/s43044-023-00359-0.

## Background

### Introduction

There has been an uptick in the occurrence of atrial fibrillation (A-fib) cases in COVID-19 patients during the recently concluded pandemic. Out of 9574 patients, COVID-19 patients admitted to ICU in Northwell Health System in New York, A-fib developed in 1687 (17.6%) of them and these patients experienced significantly higher in-hospital mortality compared to those with sinus rhythm (55.2% vs 46.8%) [1]. In a retrospective cohort study, it was revealed that the odd ratio of developing A-fib in COVID-19-positive patients is higher than that in COVID-19-negative patients (1.19 95%:1.00, 2.41) and pre-pandemic patients (1.57 95% CI (confidence interval):1.23, 2.00) [2]. It is well known that cardiovascular risk factors such as high blood pressure, diabetes and obesity increase the propensity to develop atrial fibrillation [3]. Clinicians witnessed the increased risk for developing of cardiac arrhythmias in COVID-19 patients and this propensity increases substantially in those with higher CHA(2)DS2-VASc score [4]. These findings highlight the fact that the viral infection can alone function as an independent risk factor for developing cardiac arrhythmias and associated worse clinical outcomes. The ability of COVID-19 virus to provoke a wide variety of cardiac arrhythmias had baffled and perplexed the entire clinical and research community. Analysis of cardiovascular outcomes in 153,760 older while male patients utilizing US Veteran Health System from March 1, 2020, to January 15, 2021, revealed 19.86 incidents of dysrhythmias, 10.74 incidents of atrial fibrillation, 23.48 incidents of Major Adverse Cardiovascular Events (MACE) and 4.03 incidents of stroke for every 1000 patients with COVID-19 diagnosis [5]. The precise etiology for occurrence of atrial fibrillation in COVID-19 patients is currently obscure. Even though there is dearth of research studies in this regard, some researchers tried to implicate cytokine storm, NLRP3 inflammasome, hypoxemia, endothelial dysfunction, electrolyte disturbances, microvascular inflammation, platelet activation, fibrin deposition and activation of sympathetic nervous system for inciting atrial fibrillation in COVID-19 patients [6, 7]. Since this is relatively a new clinical phenomenon, improved understanding of crucial etiological factors and their interplay at the cellular level particularly in atrial cardiomyocytes, endothelial cells and pericytes would yield valuable information regarding the probable pathophysiological mechanisms leading to generation of atrial substrate. Moreover, the clinical presentation of A-Fib is very vague and clinicians managing in COVID-19 patients admitted to ICU should be more vigilant for this clinical entity. This is very critical because it usually requires prompt diagnosis and management for preventing poor cardiovascular outcomes and its associated complications. It is important to understand that COVID-19 and A-fib is a lethal combination because they together increase the risk of pulmonary embolism, stroke and venous thromboembolism [7]. Sometimes, it is quite possible atrial fibrillation in COVID-19 patients can degenerate into ventricular fibrillation and eventually cause cardiac arrest and sudden death. In this review, which is presented we summarized the epidemiology, pathophysiological mechanisms, clinical symptomatology, diagnosis, therapeutic options and complications of COVID-19-induced A-fib. Furthermore, we discussed extensively the scope of future research that can be performed in the cell culture and animal models for unraveling the potential mechanisms for generation of atrial substrate, which can induce atrial fibrillation in COVID-19 patients. We hope that this review will fill the knowledge gap in understanding of pathophysiological mechanisms of COVID-19-induced atrial fibrillation and would jumpstart basic science and clinical research studies in this regard. Any useful preliminary information gleaned from these studies can be exploited for drafting novel therapeutic modalities for decreasing mortality and morbidity of this clinical entity.

### The spectrum of cardiac arrhythmias in COVID-19 patients admitted to hospital

The prevalence of arrhythmias in COVID-19 patients admitted to the hospital is around 18% and 15% during admission and discharge, respectively [8]. The incidence of ECG abnormalities in COVID-19 patients includes sinus tachycardia (85.5%), atrial fibrillation (10.5%), atrial block (2%), atrial flutter (1%) and junctional rhythm (0.5%) [8]. An observational study conducted on COVID-19 patients admitted to the hospital revealed the incidence of arrhythmias as follows; premature ventricular complexes (28.7%), non-sustained ventricular tachycardia (15.4%), sustained ventricular tachycardia (1.4%) and ventricular fibrillation (0.7%) [9]. In this observational study, the overall mortality of COVID-19 patients which were followed up for 23 days was approximately 25% [9]. The spectrum of arrhythmias in 700 COVID-19 patients admitted to a hospital and serially followed up for 2.5 months, include cardiac arrest (9), atrial fibrillation (25), clinically significant bradyarrhythmias (9), non-sustained ventricular tachycardia (NSVT) (10) [10]. In this previous study, the in-hospital mortality was highest in the patients developing cardiac arrest (OR 20.47; 95% CI 5.19–80.69) followed by atrial fibrillation (OR 6.73; 95% CI 2.52–17.98) [10]. Overall prevalence of atrial fibrillation in patients with COVID-19 without and with previous history of cardiovascular diseases is around 19–21% and 36%, respectively [6].

### Epidemiology of COVID-19-induced atrial fibrillation

Atrial fibrillation is the most common cardiac arrhythmia in COVID-19 patients. The incidence of A-fib in COVID-19 patients without and with cardiovascular abnormalities is approximately 19–21% and 36%, respectively [6, 11]. The incidence tends to be even higher (23–33%) in critically ill COVID-19 with ARDS (Acute Respiratory Distress Syndrome) or sepsis admitted ICU (Intensive Care Unit) [6]. Out of 30,999 COVID-19 patients admitted to 120 medical institutions in USA, A-fib developed approximately 1517 patients (5.4%) who have associated cardiovascular risk factors [3]. Retrospective analysis of 78,725 COVID-19 patients with comorbidities in Mass General Brigham Health System revealed that the odds of developing A-fib is 1.18 times in COVID-19 patients compared to non-COVID-19 patients [2]. Meta-analysis of studies performed until February 2021 to assess the prevalence of A-fib in COVID-19 patients demonstrated that it is 2.5 times more common older population (> 60 years) compared to younger population [12].

Demographically, the incidence of A-fib is more common in Europeans (15%) followed by Americans (11%), Asians (6%) and Africans (2%) in COVID-19 patients [12]. Retrospective analysis of 3970 COVID-19 patients diagnosed between February 4 to April 22, 2020, indicated that the prevalence of A-Fib is 10% and 4% in COVID-19 patients with and without history of atrial arrhythmias, respectively [13]. Another retrospective analysis of aged population (> 60 years) from multicenter registry of Italian and Norwegian societies of Gerontology and Geriatrics unveiled that the incidence of A-fib is around 21.8% and it more prevalent in those with higher CHA_2_DS_2_-VASc score [14]. Multicenter cohort study of COVID-19 patients diagnosed with PCR from March 2020 to December 2021 from 17 hospitals affiliated with University of California indicated that the risk of A-fib (10.85%) is lower in COVID-19 patients than in non-COVID-19 patients (14.16%) [15]. Single-center retrospective analysis of 492 patients admitted to Bahrain Defense Force COVID-19 ICU from April 2020 to December 2020 revealed higher risk of developing A-fib in COVID-19 group (66.7%) compared to control group (17.1%) [16].

## Main text

### Underlying mechanisms hypothesized for occurrence of COVID-19-induced A-fib

**Fig. 1:**
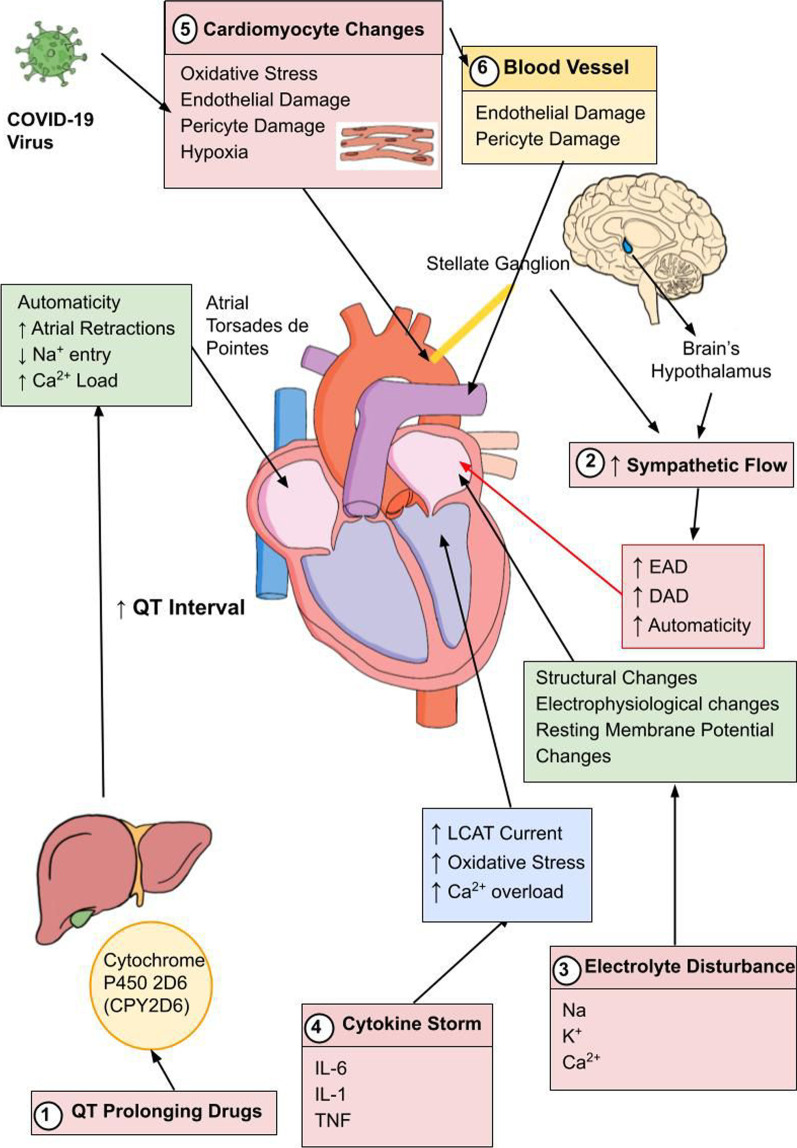
Extracardiac factors that are implicated in manifestation of Atrial Fibrillation in COVID-19 patients

**Fig. 2:**
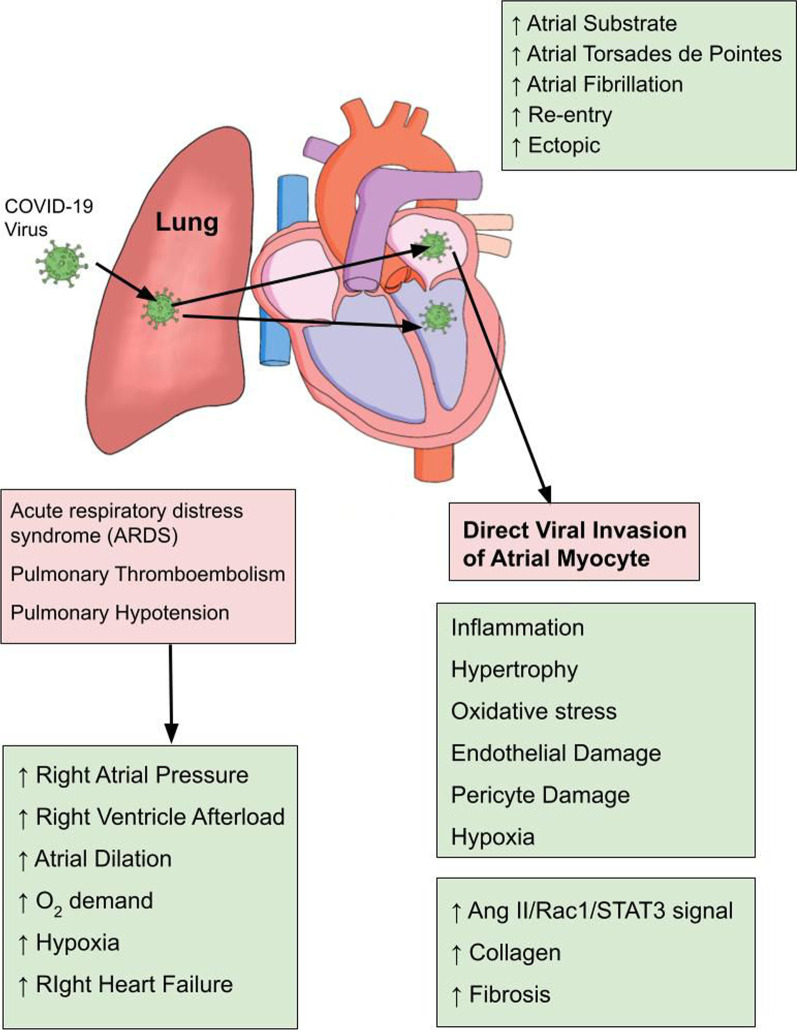
Cardio-pulmonery effects of COVID-19 underlying the development of Atrial Fibrillation

Although atrial fibrillation is one of the most common arrhythmias in COVID-19 patients, its exact mechanism is not elucidated definitely yet. Nevertheless, researchers hypothesized some speculative mechanisms for its occurrence which can be summarized as downregulation of angiotensin-converting enzyme 2 (ACE2), CD-147, sialic acid-spike protein interaction, cytokine storm, endothelial damage, electrolyte disturbances, hypoxemia and hyperactive sympathetic nervous system [6]. Loss of surface ACE2 from atrial myocytes and vascular endothelial cells due to its internalization secondary to COVID-19 binding causes deprivation of its protective effect resulting in various cellular alterations such as hypertrophy, increased vascular permeability, endothelial dysfunction, inflammation, and fibrosis. COVID-19-induced myocardial inflammation is a clinical entity that has been widely documented in previous studies [17, 18]. This myocardial inflammation can entail activation of downstream signaling pathways (STAT3/Rac3/Angiotensin-II) which would provide basis for increased collagen synthesis, atrial fibrosis, structural remodeling and atrial substrate formation for subsequent development of atrial fibrillation [19] (Fig. 1). Pulmonary thromboembolism and pulmonary hypertension are well known reported complications of COVID-19 patients [20, 21]. These clinical abnormalities provoke several pathological derangements ranging from accentuated intra-atrial pressures, elevated right ventricular afterload, increased myocardial oxygen demand, hypoxia, and massive atrial dilation all of which in combination increase the risk for developing atrial fibrillation [22] (Fig. 2).

Upon entering into the atrial myocytes, it undergoes replication and provokes multifarious pathological derangements that underlie the susceptibility to cardiac arrhythmias. First, it can impair the intercellular communication between adjacent cardiomyocytes by downregulating the expression of connexins (Cx40 and Cx43), thus leading to derailed impulse conduction and protracted cardiac cycle duration [23]. Second, it can cause wreckage in the functioning of intracellular calcium handling proteins such as ryanodine receptors (RyR), sarco-endoplasmic reticulum calcium ATPase (SERCA) and phospholamban (PLB) leading to increased calcium release and intracellular calcium accumulation [23]. Third, it can facilitate inward depolarizing (*I*_CaL_, *I*_Na_) currents and the same time antagonism of outward repolarizing currents (*I*_to_, *I*_Ks_, *I*_Kr_). Fourth, direct invasion of cardiomyocytes can also lead to myocardial inflammation due to combined effect of inflammatory infiltrate, immune response and cytokines [23] (Fig. 1). Lastly, viral involvement of neighboring cardiac fibroblasts can lead to activation of fibrogenic pathways, extracellular matrix formation and deposition of fibrogenic tissue in the atrial myocardium [23]. These pathological derangements cultivate a viable breeding ground for initiation and sustaining of atrial fibrillation in COVID-19 patients.

Due to disturbance of the delicate balance between Th1 and Th2 lymphocytes, cytokine storm is unleashed with production of pro-inflammatory cytokines takes place [6]. Systemic inflammatory response syndrome along with upregulation of cytokines such as IL-6, TNF and IL-1 can be responsible for prolongation of ventricular action potential [24]. These cytokine induced cellular events in atrial myocytes can incite atrial fibrillation. One the important mechanisms suggested for cytokine induced atrial fibrillation include increased intracellular calcium overload and elevated *I*_cal_ (L-type calcium current) [23] (Fig. 1). Furthermore, oxidative stress secondary to inflammation within the atrial myocyte can be responsible for initiating and sustaining atrial fibrillation. Sustained inflammatory state in COVID-19-induced atrial fibrillation documented by marked upregulation of IL-6, CRP (C-reactive protein) and plasma viscosity provides a forewarning for occurrence of thromboembolism and stroke in these patients [7, 19, 25, 26].

Electrolyte disturbances (hypokalemia, hyponatremia and hypocalcemia) are considered as one of the most important risk factors for development of tachyarrhythmias including atrial fibrillation [27] (Fig. 1). The occurrence of electrolyte disturbances in COVID-19 patients is explained by acute renal failure, gastrointestinal disturbances, activation of renin angiotensin system and inflammation [22]. Hyponatremia and hypokalemia tend to induce atrial fibrillation via altering the electrophysiological properties of sino-atrial node (decreased beating rate) and pulmonary vein (attenuated burst firing) [28]. Hypokalemia leads to increased atrial arrhythmogenic potential due to sway on resting membrane potential with changes ranging from fastened depolarization, predominance of hyperpolarization state to increased atrial resting membrane potential [27]. These atrial cellular changes leads to incitement of ectopic beats and reentry phenomenon, which combined increases the propensity to develop atrial fibrillation [27]. Hypocalcemia induced atrial fibrillation can be attributed to structural changes, alteration of calcium cycling and revamping the electrical activity [27].

QT prolongation which can be due to virus itself or due to administration of QTc prolonging drugs for managing these patients can be a likely predisposing factor for initiation of cardiac arrhythmias in these patients [24] (Fig. 1). COVID-19 virus might alter the metabolizing activity of the liver enzymes (cytochrome P450), thereby increasing the half-life of QT prolonging drugs that are currently administered to these patients [23]. Studies have shown that patients with long QT interval syndrome have an increasing proclivity to develop atrial fibrillation [29]. Increased propensity of atrial fibrillation in long QT interval syndrome might be secondary to prolonged atrial muscle refractoriness, delayed sodium entry into atrial myocytes, increased intracellular calcium load and automaticity [30]. These cellular abnormalities can pave the way for occurrence of atrial torsades de pointes (polymorphic atrial tachycardia with undulating P-wave) which has the potential to degenerate into atrial fibrillation [30]. Antimalarial (Chloroquine) and anti-viral (Ritonavir) drugs usually prescribed for managing COVID-19 infections indirectly prolong the half-life of QT prolonging drugs via inhibition of cytochrome P450 2D6 enzymes in the hepatic tissues [31]. These QT prolonging drugs might also have a collateral effect of attenuating HERG K^+^ channels, thereby resulting in lengthening of ventricular depolarization [31]. QT prolongation is associated with systemic inflammatory response characterized by IL-6 upregulation and administration of IL-6 receptor blocking antibody (Tocilizumab) had yielded successful outcomes in decreasing the risk of QT interval related tachyarrhythmias including atrial fibrillation [31]. Furthermore, among patients with atrial fibrillation who underwent catheter ablation, the presence of longer QTc can be regarded as an independent risk for developing future stroke and heart failure [32].

COVID-19-induced kick starting of central inflammatory reflexes and peripheral stellation ganglia activation can provoke increased sympathetic outflow to the atrial myocardium [23]. Enhanced sympathetic outflow leads to early after-depolarization (EAD), delayed after-depolarization (DAD) and enhanced automaticity, thereby increasing the ectopic activity in atrial myocytes [33] (Fig. 1). The other mechanisms proposed for establishment of A-fib substrate with sympathetic stimulation include calcium overload, over expression of hypertrophic and pro-fibrotic genes, larger window currents, augmentation of slow delayed-rectifier K^+^-current (*I*_Ks_) and attenuation of inward-rectifier K^+^-current (*I*_K1_) [33] (Fig. 1). Moreover, increased expression of fibrogenic genes would probably lead to excess deposition of fibrous tissue resulting in segregation of adjacent atrial myocytes [19]. This uncoupling of atrial myocytes would cause inter-atrial conduction disturbances, thus providing a hotbed for subsequent development of atrial fibrillation [19].

### How about the cellular mechanisms for A-fib from other inflammatory involvement of heart muscle in general (e.g., viral, pneumonia or autoimmune)

Major aberrations instigated by viral infections that predispose to occurrence of A-fib can be classified into impairment of atrial electrophysiology and structural remodeling [34]. In this regard, atrial fibrosis, action potential shortening, breakdown of intercellular connections and alteration of calcium handling are speculated to be important changes that are operational for inciting atrial fibrillation. Initiation, maintenance and recurrence of A-fib with viral and systemic inflammatory diseases can be associated with presence of significant biomarkers in the blood such as MCP-1 (Monocyte chemoattractant protein-1), NLRP3 and pro-inflammatory markers (IL-1, IL-2, IL-6, IL-8 and IL-12 [34].

In nonvalvular A-fib secondary to infectious inflammatory etiology, researchers demonstrated higher levels of TLR-2 (Toll-like receptor-2) receptors and IL-6 compared to those with sinus rhythm [35]. Influenza infection increases the risk of developing A-fib and flu vaccination tend to downregulate this risk by 18% [36]. Influenza seems to incite A-fib through direct cardiomyocyte invasion, pro-inflammatory cytokine production and increased sympathetic tone, all of which are instrumental in instigating electrophysical changes necessary for generating arrhythmogenic foci [36, 37]. Influenza A virus infected hearts exhibit altered cardiac proteome which specifies high stress and low energy state [38]. Cardiomyocytes infected with influenza A virus demonstrated oxidative stress, mitochondrial damage, decreased phosphorylation, necroptosis and cell death [38]. Community acquired pneumonia tend to cause A-fib through multiple mechanisms including prothrombotic state, myocardial injury, myocardial ischemia, sepsis, pericarditis, myocarditis, impaired gas exchange and disorganized sympathetic–vagal balance [39]. In patients with gingivitis, oral bacteria tend to be arrhythmogenic through various pathways ranging from secretion of inflammatory mediators, molecular mimicry, overactivation of autonomic nervous system, direct invasion to secretion of bacterial toxins [40].

Autoimmunity favors the production of auto-antibodies against myosin, M2 receptors, Na+/K+ pump, heat shock proteins and β-adrenergic receptors [41–46]. These immune complexes in the atrial myocardium will subsequently prime the atrial myocardium to become a fertile ground for generation of A-fib [47, 48]. Moreover, autoimmune vasculitis characterized by inflammation and fibrinoid necrosis of vessel walls occurs globally in all the systemic tissues, a critical factor implicated in the pathogenesis of atrial fibrillation [47].

### Risk factors

Previous studies had shown that AF is more likely to develop in COVID-19 patients compared to non-COVID-19 patients (Odds > 1.19) and pre-pandemic patients (Odds > 1.57) [2]. Some of the risk factors that are hypothesized for increasing the propensity of developing atrial fibrillation in COVID-19 patients include mitral valve disease, heart failure, history of myocardial infarction, renal failure, COPD, obesity, diabetes, hypertension, peripheral vascular disease, hyperlipidemia, smoking and history of stroke [2]. In COVID-19 patients admitted for hospital due to the presence of comorbidities, the risk of developing AF is higher with the presence of in-hospital events such as ICU admission (64.5%), mechanical ventilation (MV) (47.6%), MV with mechanical circulatory support (7.7%), MV with vasopressors (31.8%), cardiac arrest (9.8%), myocardial infarction (13.8%), deep vein thrombosis (8.2%), stroke (4.4%), clinically significant bleeding (9%), corticosteroids (60.2%), renal replacement therapy (13.1%) and remdesivir (13.3%) [3]. The presence of other risk factors such as acute respiratory distress and sepsis is bound to accentuate the tendency to develop by AF by 12–20% [3]. Severe COVID-19 patients who are admitted to ICU are more to develop hypotension, hypoxia, and elevated pressures in right and left cardiac chambers that increases the predilection for having AF. Studies demonstrate AF is less likely to develop in black patients compared to non-Hispanic whites even though they tend to have more cardiovascular risk factors such as diabetes, hypertension and obesity [3]. Gender differences were noted with males tend to have to more proclivity to develop AF compared to females which can be partially explained by effect of male hormones, adipose tissue and body mass index [3].

### Summary of Clinical cases reported for COVID-19-induced A-fib

The clinical presentation of atrial fibrillation can be varied with non-specific signs and symptoms. The following table enumerates the age, clinical symptoms, signs, diagnostic tests, treatment and prognosis in patients presenting with COVID-19-induced atrial fibrillation.

Atrial fibrillation (A-fib) is characterized by an irregular and rapid heart rate, which can lead to various symptoms and complications. Case studies suggest that the most common symptoms observed among A-fib patients were palpitations, shortness of breath, and fatigue. Many patients also presented with altered mental status that could be a result of the atrial fibrillation. Other less frequently reported symptoms included dizziness, chest pain and syncope. Patients often presented with elevated heart rates (mean of 120 bpm) and irregular rhythms on physical examination. EKG results showed consistent evidence of A-fib in all cases, with some patients also exhibiting additional abnormalities such as atrial flutter, ST-T wave changes and ventricular ectopy.

**Table:** 

Cases	Demographics	Clinical symptoms	Signs	Diagnostic tests	Treatment and prognosis	Prognosis	References
1	73-year-old female Caucasian	Altered mental status, panic attack with palpitations, nausea, numbness, tachypnea, heavy breathing, dry cough, and chest discomfort	**BP** 137/71 **O2 sat** 83% **Tem**:99.7°F **HR** ~ 115 **RR** ~ 33 Tachycardia, Irregular rhythm Pulse deficit Bibasilar crackles	**CXR**: Diffuse pulmonary edema, bilateral interstitial infiltrates, and hazy opacities **ECG**: atrial fibrillation with RVR **CT Scan**: multiple ground glass opacities predominantly in peripherally and posteriorly	Supportive care, Dexamethasone, Hydroxychloroquine, Remdesivir, Anticoagulation, azithromycin	Clinical recovery to sinus rhythm	[49]
2	66-year-old female	On 6th day of COVID-19 related admission patient presented with tachycardia and palpitations	**HR**: 160 bpm	**EKG** showed atrial fibrillation with rapid ventricular response	Metoprolol Diltiazem Carvedilol	Clinical recovery to sinus rhythm	[50]
3	66-year-old male	Sudden onset of palpitations and worsening dyspnea	**HR**: 186 **BP**: 134/80	**EKG** showed irregular, narrow-QRS complex tachycardia without P waves	Amiodarone External Electric shock 260 J	Brainstem acute ischemic stroke, ventricular fibrillation and cardiac arrest	[51]
4	90-year-old African American female	Altered mental status from welfare check	**BP** 141/78 **Tem**:97.9°F **HR** ~ 140 **RR** ~ 44	**ECG**: atrial fibrillation with RVR Echo: Ejection fraction 65–70% with grade I diastolic dysfunction	Intubation, supportive care, Hydroxychloroquine azithromycin	Clinical recovery to sinus rhythm	[52]
5	50-year-old male	Pain on the posterior aspect of the right lower leg	**BP** 129/76 **Tem**:98.9°F **HR** ~ 78 **RR** ~ 18	**EKG** showed irregular heart rate with new-onset AF **CT scan extremities**: partial right popliteal block **CT abdomen**: Left kidney lower pole infarction **MRA**: Right occipital lobe subacute infarcts	Inpatient: Azithromycin, oseltamivir, paracetamol, Vitamin C, zinc sulfate, Hydroxychloroquine Bisoprolol and Omeprazole. Outpatient: Direct acting oral anticoagulant (DOAC), atorvastatin, aspirin and bisoprolol Follow-up with AF clinic for electric cardioversion	Clinical recovery to sinus rhythm	[53]
6	72-year-old female	Altered mental status	**HR** ~ 133 Tachypnea Leukocytosis Increased lactate	**EKG** New-onset AF with RVR **Troponins**: > 20,000 ng without ST and T elevations suggesting acute cardiac ischemia	Cardizem drip followed by oral amiodarone and metoprolol	Clinical recovery to sinus rhythm	[54]
7	57-year-old male	Palpitations and progressive dyspnea	**BP** 117/97 **O2 sat** 97% **HR** ~ 152 **RR** ~ 14 Irregularly irregular puse Bibasilar fine crepitations Elevated JVP	**EKG** New-onset AF Echo: LVEF 20% and MR Cardiac MRI: biventricular edema, dysfunction with LVEF 30%. Severe myocarditis	Intravenous diuretics, rate control agents, anticoagulation, ACE inhibitor and mineralocorticoid	Clinical recovery. Cardiac MRI at 3-month interval planned with outpatient follow-up	[55]
8	78-year-old Caucasian male	Altered mental status, panic attack, palpitations, nausea, numbness, tachypnea, heavy breathing, dry cough and chest discomfort	**BP** 137/71 **O2 sat** 83% **Temp** 39.7C **HR** ~ 115 **RR** ~ 33 Irregular rhythm Bibasilar crackles Pulse deficit	**EKG** AF with RVR **Anion gap**: abnormal Metabolic alkalosis Echo: borderline abnormal LVEF (50–55%), LV diastolic dysfunction, mild pericardial effusion, moderate septal hypertrophy, mild dilated LV (LVEDD: 58.9 mm and LVESD: 40.7 mm) and restrictive LV filling pattern	ICU admission, intubation, vasopressor, supportive care, dexamethasone, hydroxychloroquine, remdesevir, IV Ibutilide, anticoagulation and azithromycin	Normal sinus rhythm was restored two days prior to extubation	[56]
9	72-year-old male	Severely hypoxic patient (Sp02 65%)	Irregular heart rhythm, exhausted, perspiring, bilateral medium crackles,	**EKG:** AF **Echo**: Globally reduced LV systolic function, EF 30%	Intubation, Non-adrenaline, Dobutamine, Volume resuscitation, Argipressin, Dexamethasone, Landiolol, amiodarone, digitoxin, ivabradine and Levosimandan	Pericardial tamponade Cardiogenic shock Death	[57]
10	18 y male	Refractory hypotension, blurry vision, eye redness, nausea, vomiting, cheat pain, nausea, vomiting, syncope, dizziness and refractory hypotension	**BP** 80/40 **HR** ~ 120 Hepatomegaly Injected conjunctiva Erythematous tongue Blanching rash on palm and wrists	**EKG:** AF, wide complex tachycardia **Echo**: mild pericardial effusion	Cardioversion 100–150 J Bolus of Amiodarone Low dose epinephrine	Normal sinus rhythm is restored with complete clinical recovery	[58]
11	15-year-old African American female	Headache, vomiting and fatigue	**BP** 70/90 **Temp** 102.8 F **HR** ~ 150	**Echo:** Severe LV dysfunction without atrial or ventricular dilation **EKG**: AF with RVR	IV saline, milrinone, epinephrine, IV immunoglobulin, IV methyl prednisolone, subcutaneous LMW heparin, IL-1 receptor antagonist (Anakinra). Cardioversion (50 J) and Amiodarone	Normal sinus rhythm is restored with complete clinical recovery	[59]

**Table:** 

Demographics	Clinical symptoms	Signs	Diagnostic tests	Treatment and prognosis	Prognosis	References
66-year-old female	COVID-19 patient presented with tachycardia and palpitations on the 6th day of admission	**HR**: 175 bpm	**EKG**: Irregularly irregular rhythm with absence of P waves AF with RVR Echo: Preserved ventricular fraction with no other abnormality	IV Metroprolol, IV diltiazem and Carvedilol 30 mg every 6 h	Restoration of sinus rhythm with clinical recovery	[50]
84-year-old male	Shortness of breath, fever and generalized weakness	**BP** 136/75 **O2 sat** 99% **Temp** 36.8C **HR** ~ 68 **RR** ~ 23	**EKG:** Atrial flutter with varied AV block **Chest CT:** no pulmonary embolism **Echo:** normal LV and RV function and size, mild TR and mild atrial enlargement	IV dexamethasone and oral anticoagulation	Restoration of sinus rhythm with clinical recovery	[60]
29-year-old male	Fever, chills, sweats, palpitations, and shortness of breath	**BP** 127/72 **O2 sat** 91% **Temp** 38.05 C **HR** ~ 131 **RR** ~ 28	**EKG:** Irregularly irregular RR interval, no distinct P waves **Chest CT:** no pulmonary embolism **Echo:** Mildly dilated LV, severe global LV dysfunction, moderate RV hypokinesis, severe LA enlargement	IV Cardizem, Metrprolol and flecainide and oral anticoagulation with Eliquis	Restoration of sinus rhythm with clinical recovery	[60]
46-year-old black male	Fever, cough and shortness of breath	**SBP** 140 **O2 sat** 88% **HR** ~ 142 **RR** ~ 28	**EKG:** Atrial flutter, 2:1 AV block and ST-T changes **Echo:** preserved LV ejection fraction, no regional wall motion abnormalities	Amiodarone, digoxin bolus, -blockers, cardioversion 100 J and oral anticoagulation	Restoration of sinus rhythm with clinical recovery	[61]
59-year-old male	Palpitations, exertional dyspnea and fatigue	**BP** 134/90 **O2 sat** 96% **Temp** 37.3 C **HR** ~ 136 **RR** ~ 16	**EKG:** Atrial fibrillation with PR 134 bpm and abnormal R wave progression **Chest CT:** unremarkable Echo: moderate LV hypertrophy, LV ejection fraction 60%. Normal LV and LA size	IV followed by oral Diltiazem and cardioversion	Restoration of sinus rhythm with clinical recovery	[62]
56-year-old Hispanic male	Malaise and palpitations. Presented to the urgent care previously with fever, chills, night sweats, headaches and palpitations	**BP** 124/100 **O2 sat** normal **Temp** 38.8 C **HR** ~ 130–140 **RR** ~ 16	**EKG**: Atrial fibrillation with PR 131 bpm along with evidence of LV hypertrophy	IV Diltiazem and fluid bolus	Restoration of sinus rhythm with clinical recovery	[62]

### Clinical management

Atrial fibrillation is regarded as an important risk factor for Major Adverse Cardiovascular events (MACE) such as all point death, heart failure, myocardial infarction and stroke [63]. According to a study done by Cutler et al. both previous and current A-fib in COVID-19 patients is associated with worse clinical outcomes [63]. Accordingly, efforts should be made to carefully diagnose, monitor and treat A-fib in a prompt manner for preventing adverse consequences in COVID-19 patients. Treatment of new-onset atrial fibrillation in COVID-19 is necessary for restoring the sinus rhythm as well as for optimizing the cardiac output. This can accomplished with therapeutic modalities including rate and rhythm control medications [6, 64]. Another important aspect of management of atrial fibrillation is preventing the future risk of thromboembolism with the administration of preventive anticoagulant therapy [6, 64]. Since COVID-19 infections are associated with many complications such as electrolyte disturbances (hypokalemia, hyponatremia, hypomagnesemia and hypocalcemia) and hypoxia that can precipitate AF, efforts should be made to address these abnormalities in addition to offering therapeutic medications [6, 65]. The presence of hemodynamic stability COVID-19 patients with new-onset A-Fib is one of the most critical determining factor for administration of cardioversion [6]. In patients without any cardiovascular instability, it is would be prudent to administers rate controlling medications including Class II agents (β-blockers—Carvedilol, Metoprolol and Esmolol), Class IV agents (Calcium Channel blocker [CCBs] agents—Diltiazem and Verapamil) and Digoxin [6, 64, 65]. In the contrary, presence of hemodynamic instability with COVID-19 patients with A-Fib warrants delivery of cardioversion along with rhythm control agents including IA (Procainide, Disophyramide and Quinidine), IC (Flecanide and Propafenone) and III class agents (Amiodarone, Dronedarone, Dofetilide, Ibutilide and Sotalol) [6, 64, 65]. Anticoagulation in COVID-19 patients with atrial fibrillation is accomplished by drugs including Direct Xa factor inhibitors (Apixaban, Rivaroxaban and Edoxaban), Direct thrombin inhibitors (Dabigatran), Vitamin-K antagonist (Warfarin) and heparin [64, 65]. The preferred choice of rate control agents depends on the presence or absence of congestive heart failure (CHF) [64]. In the absence of CHF, rate control is achieved via usage of β-blockers, CCBs, digoxin and amiodarone [64]. On the other hand, the presence of acute decompensated CHF in critically ill COVID-19 warrants administration of digoxin or amiodarone [64]. The choice of rhythm control drugs in COVID-19 patients depends on the presence of absence of structural heart disease [64]. In the absence of structural heart disease, Amiodarone, Flecainide, Ibutilide and Sotalol are the drugs of choice for achieving rhythm control [64]. In the presence of myocarditis or CHF or coronary artery disease, rhythm control is attained through administration of Amiodarone or Dofetilde or sotalol [64]. The role of catheter ablation in COVID-19 patients with A-fib is controversial although it is usually reserved in patients experiencing significant heart failure, frequent emergency department visits and those having significantly higher risk of thromboembolism [6].

**Fig. 3:**
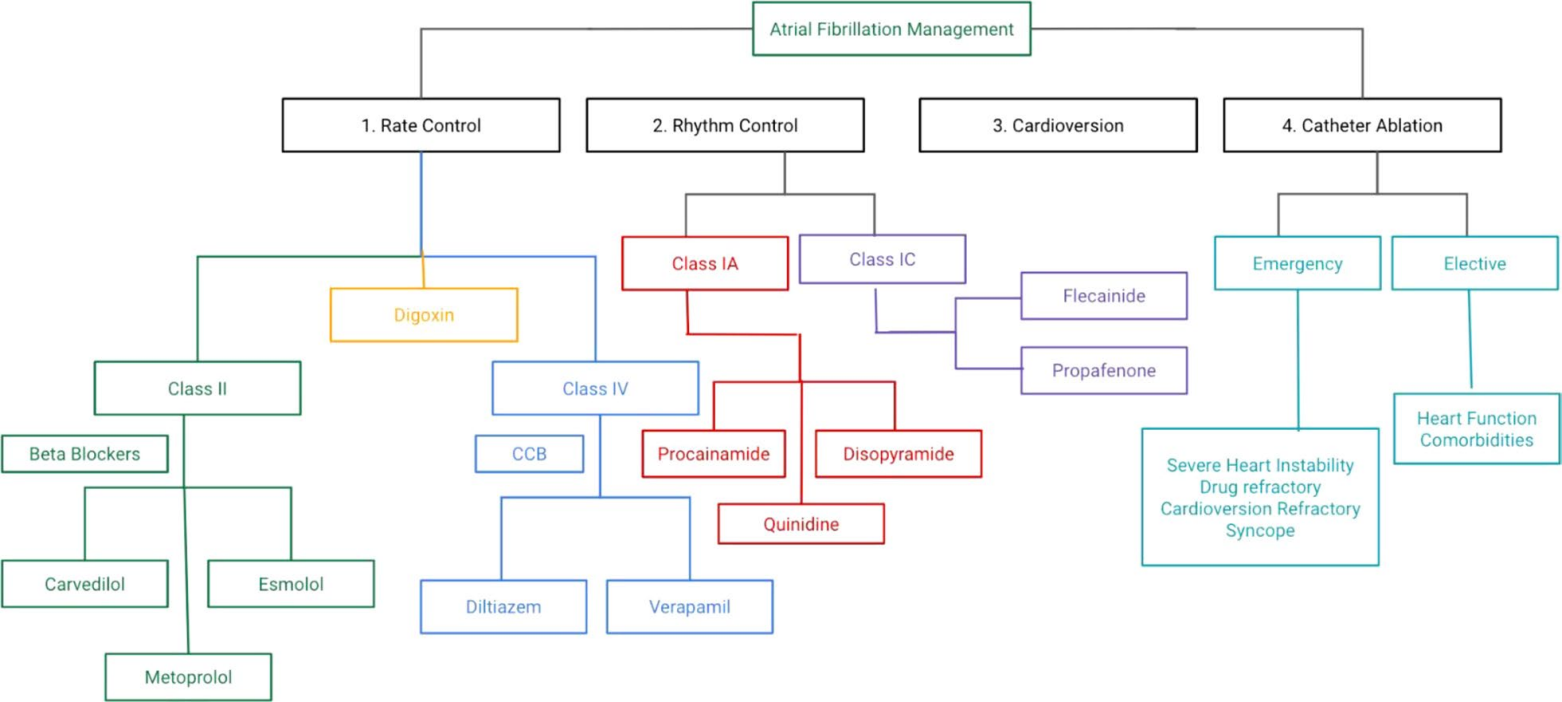
Treatment options in management of COVID-19 induced A-fib

Emergent need of catheter ablation is warranted in A-Fib with severe symptoms and hemodynamic instability or collapse, A-Fib refractory to cardioversion/drugs/rate control, refractory A-Fib with several emergency department visits and A-fib with syncope or cardiac arrest [65, 66]. On the contrary, elective catheter ablation is considered in COVID-19 patients with stable heart function, comorbidities and no future risk of hospitalization secondary to A-Fib [66]. Comparison of preoperative practices (transesophageal echocardiography) and acute procedural outcomes of catheter ablation during and before COVID-19 pandemic revealed lesser rate of COVID-19 transmission, reduced complication rate and higher same day discharges in the 2020 cohort compared to 2019 cohort. These findings emphasize that complex electrophysiological procedures can be safety administered to high-risk COVID-19 patients with atrial fibrillation results in optimal clinical outcomes while maintaining the quality of care [67]. Analysis of 269 patients who underwent catheter ablation for arrhythmia in COVID-19 patients from January 1, 2020, to March 24, 2020, revealed arrhythmia-free survival in 95.9% patients, no COVID-19 infections reported during brief hospitalization period (3–5 days), and no COVID-19 infections during 3-month follow-up [68]. Although physician’s risk assessment is vital in identifying the COVID-19 patients suitable for catheter ablation, application of quality of life AFEQT questionnaire (Atrial Fibrillation Effect on Quality of Life) was more sensitive in providing insights into their symptom perception, functional impairment and treatment concerns as well in recognizing the high-risk A-fib patients preferable for the procedure during hospital admissions [69]. The treatment modalities used for the management of COVID-19-induced A-Fib is summarized in Fig. 3.

### Drug–drug interactions encountered in management of COVID-19 patients with A-Fib

The medications used to control A-Fib should be used cautiously as they can interact with therapeutic drugs used to neutralize the COVID-19 virus and attenuate the associated systemic inflammation. The interactions can be classified as (a) serious interactions needing to modify the drug regimen, (b) moderate interactions necessitating close monitoring and follow-up, (c) minor interactions that required caution while administering drugs and (d) no interactions where medications can be given with no follow-up. These interactions are summarized in the Additional file 1: Table S1.

### Thrombosis and systemic thromboembolism in COVID-19-induced A-Fib

**Fig. 4:**
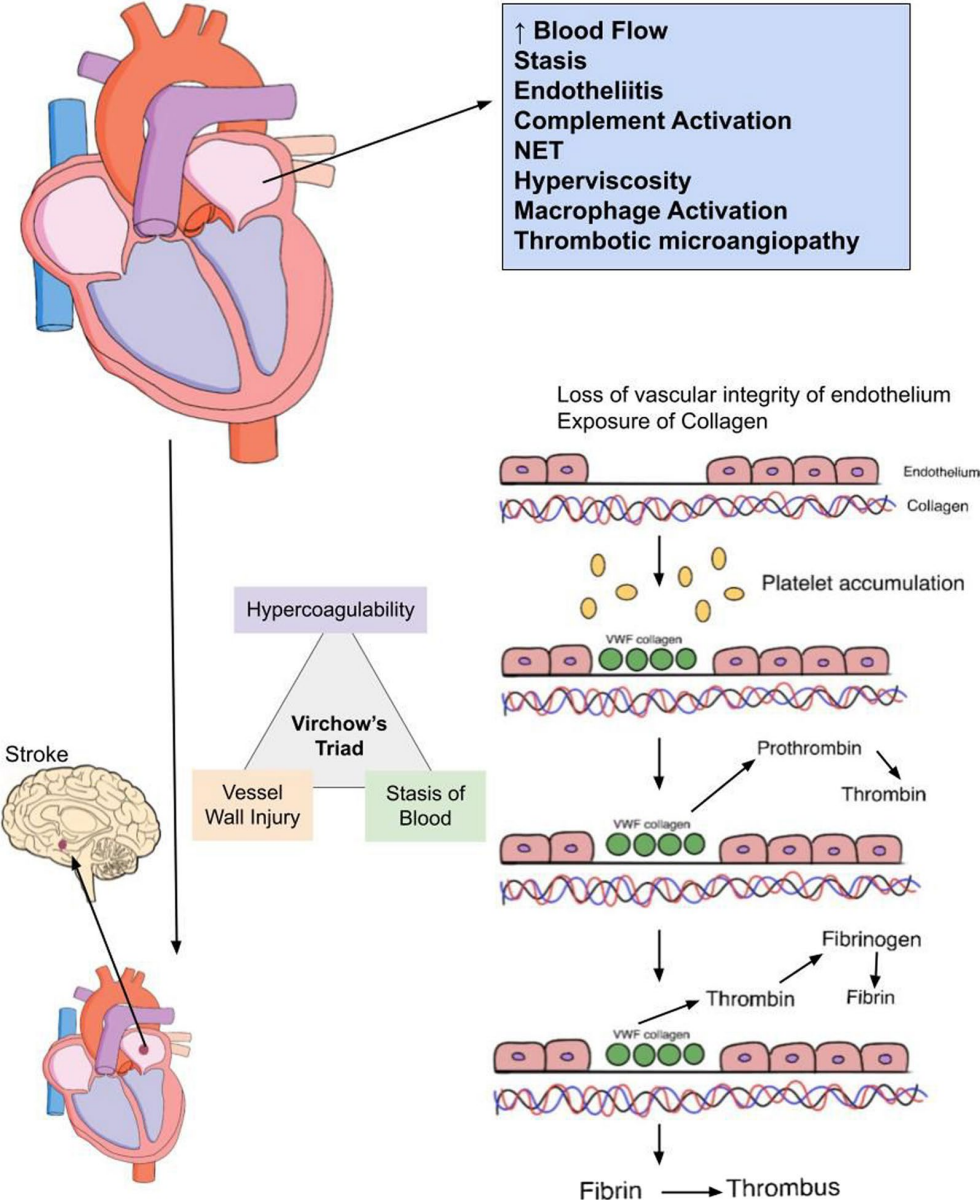
Mechanism of thrombogenesis in COVID-19 induced Afib

The presence of COVID-19 infection along with A-fib substantially increases the thrombogenic risk. The underlying risk factors that increased the risk of thrombogenic risk in COVID-19 patients can range from cytokine storm, microvascular inflammation, increased D-dimer levels, upregulated factor VIII levels, loss of endothelial integrity, platelet activation to deposition of platelets in multiple organs [7]. The initial precipitating event that incites thrombogenesis in A-Fib is increased velocity and blood flow through the enlarged left atrium. This will set in motion cascade of events that increases the probability of developing intra-atrial thrombus and subsequent systemic thromboembolism including stroke [7]. Rapid speed and flux of blood flow provokes the dislodgement of protective glycocalyx from the vascular endothelium and exposes it to circulating platelets [7] (Fig. 4). Platelets tend to accumulate in these bared vascular endothelia by taking the assistance of von Willebrand factor and collagen [7]. This platelet accumulation generates thrombin, which will subsequently convert fibrinogen into fibrin leading to thrombus generation and stabilization [7] (Fig. 4). Other predisposing factors that are conducive to thrombus formation in atrial fibrillation include stasis, endotheliitis, complement activation, neutrophil extracellular trap formation, hyperviscosity, macrophage activation and thrombotic microangiopathy [70] (Fig. 4). Stasis, hyper-coagulable state and vascular inflammation (Virchow’s Triad) (Fig. 4) in combination are responsible for occurrence of thrombosis and embolism in COVID-19-induced AF [71]. Moderate to severe COVID-19 patients are more likely to be admitted to ICU for longer periods, thereby making them to susceptible to stasis of blood within their vasculature [71]. COVID-19 infection was shown to be associated with vasculitis of cutaneous and systemic blood vessels, a predisposing factor for subsequent development of cryptogenic stroke and thromboembolic complications in these patients [71]. Hyper-coagulable state that develops in COVID-19-induced A-Fib is attributed to cytokine storm within vascular endothelium of microvasculature within atrial myocardium [71]. These pro-inflammatory cytokines can directly stimulate the platelet activation and upregulate the synthesis of tissue factor (TF), Von-willebrand factor and factor VIII [71]. This results in increased thrombin generation and subsequent fibrin clot deposition leading to formation of thrombus within atrial chambers [71]. Atrial thrombus formation is considered a forewarning for future occurrence of thromboembolism that can manifest as stroke or transient ischemic attacks (TIA) (Fig. 4). The occurrence of atrial fibrillation in COVID-19 patients with risk factors such as diabetes, hypertension and hyperlipidemia leads to inflammation and oxidative stress within the vascular endothelium.

These pathological events drive atherosclerotic plaque formation, remodeling and endothelial damage, which ultimately leads to athero-thrombosis and thromboembolism [72]. CHADS2 scores and AF burden are two most important clinical factors that are taken into consideration while assessing the patient risk of thromboembolism [73]. The risk of stroke in patients with CHADS2 score 1 and continuous A-Fib, CHADS2 score 2 and no A-Fib burden and CHADS2 score 3 irrespective of A-Fib burden is 5%, 1% and 5% [73]. In a recent conducted ASSERT study (Asymptomatic Atrial Fibrillation and Stroke Evaluation in Pacemaker Patients and the Atrial Fibrillation Reduction Atrial Pacing Trial) assessing 2580 patients, the risk of stroke in patients with AF episodes lasting for < 17.7 h and > 17.7 h is 1.3-fold and fivefold, respectively [74]. Studies indicate that continuous A-fib episodes lasting up to 48 h are usually sufficient for thrombus formation and subsequent stroke development. These high-risk patients usually benefit from anticoagulant therapy, which reduced the risk of thromboembolism (stroke) drastically by 65% [73].

Patients having A-Fib greater than 48 h are managed with 3 weeks of anticoagulation and transesophageal echocardiography before they are subjected to cardioversion [75]. On the other hand, patients experiencing A-Fib that is acute in onset and less than 48 h can be started on anticoagulation any time before they are considered eligible for cardioversion [75]. All A-Fib patients should be considered for long term anticoagulation based on their CHADS2 score, AF burden, bleeding risk and thromboembolism risk [75].

The incidence of stroke in patients with COVID-19-induced A-Fib can range from 28 to 33% [71, 76, 77]. Ischemic stroke is more common than hemorrhagic stroke in patients with COVID-19 patients with small percentage of them presenting with intracranial herniation secondary to hematoma [71]. Retrospective analysis of COVID-19 patients revealed that ischemic stroke is the most common neuro-radiological finding (27%) followed by micro-hemorrhages (18%) [78]. Another retrospective observational study looking into risk factors, stroke characteristics and clinical outcomes in a large health care system in New York City from March 1, 2020, to April 30, 2020, revealed that atrial fibrillation/flutter, cardioembolic and cryptogenic stroke are present in 10%, 28.9 and 51.8% of COVID-19 patients [77]. In a community-based longitudinal study involving 15,000 older black patients, the occurrence of A-fib was associated with twofold increased risk of venous thromboembolism (VTE) and concurrently VTE increased the propensity of developing A-Fib by twofold [79]. In a Swedish Nationwide Registry Study, the incidence of VTE was higher in both men and women within first 30 days after A-Fib diagnosis with hazard ratios 6.64 (95% confidence interval, 5.74–7.69) and 7.56 (95% confidence interval, 6.47–8.83) and administration of anticoagulation was shown to protective against VTE and stroke [80]. VTE in the setting of COVID-19-induced A-fib might also be associated with pulmonary embolism [81]. In a recently conducted community study, it is apparent that the risk of developing pulmonary embolism (PE) is very high during first 6 months of A-Fib [79]. The mechanism of developing PE in the patients with COVID-19-induced A-Fib is not very clear although some hypothesis were proposed to explain its occurrence.

Formation of thrombus in the left atrium (LA) and left atrial appendage (LAA) can be sometimes associated with thrombus formation in the right atrium (RA) and right atrial appendage (RAA) [81]. Echocardiography studies suggest that LA and LAA (10–15%) are more likely to harbor thrombus compared to RA and RAA (0.4–7.5%) due to differences in location, anatomy and morphology [81–83]. The percentage of patients with thrombus formation in LA and RA unveiled in a previously conducted autopsy study is approximately 12.6% and 7.5%, respectively [84]. In the contrary, the approximate risk of thrombus formation in LAA and RAA is around 10.3% and 0.75%, respectively [81]. Most of the patients with diagnosed PE are routinely managed with trans-thoracic echocardiography instead of transesophageal echocardiography, which does not have the technical capability to identify a potential RAA thrombus. Thus, RAA thrombus even though it is present in minority set of patients fails to get spotted with current clinical practice guidelines. The formation of RAA thrombus poses a dangerous risk of developing PE because a wide opening of the RAA ostium may increase the odds of migration of RAA thrombus toward the pulmonary vasculature, getting caught in the venous system and eventually blocking the pulmonary outflow.

Pulmonary embolism is more likely to develop in COVID-19 patients with cardiovascular risk factors, and atrial fibrillation is the most common cardiometabolic risk factor encountered in them [85]. Activation of thrombogenic pathways globally in systemic tissues can be the underlying mechanism leading to pulmonary embolism in COVID-19-induced A-fib patients [7]. Higher incidence of pulmonary embolism in patients with COVID-19 and A-fib can be rationalized by the presence of multiple sub-cellular aberrations such as endothelial dysfunction, inflammation, platelet activation, adrenergic overstimulation and venous stasis confined to right atrial myocardium and venous blood vessels [7]. Blood clots formed in RA and venous vasculature as a results of these aberrations can embolize into pulmonary vasculature leading to pulmonary embolism. Earlier studies convey the message that pulmonary embolism was occurring at much rate in the earlier stages of COVID-19 pandemic, and this incidence was substantially reduced by inclusion of potent thromboprophylaxis drugs in COVID-19 management [86].

A retrospective cohort study conducted uncovered the fact that the presence of A-fib in patients with pulmonary embolism increased the risk of mortality at 1-month and 6-month interval [87]. On the contrary, development of pulmonary embolism in COVID-19 patients might increase the chance of developing A-fib via provoking changes including right ventricular strain/dilation, sympathetic overstimulation, myocardial ischemia and wreckage of atrioventricular (AV) node [88].

### COVID-19-induced A-fib degenerating into ventricular arrhythmias

COVID-19-induced A-Fib might secondarily precipitate ventricular arrhythmias including ventricular fibrillation (VF) and ventricular tachycardia (VT) via abbreviated AV node refractory period during exercise, sympathetic overstimulation, ventricular myopathy and genetic variations [89, 90]. In a multicenter prospective study where 204 COVID-19 patients were evaluated 3 months after diagnosis, ECG evaluation showed that atrial fibrillation, non-sustained ventricular tachycardia (NSVT) and premature ventricular contractions (PVC) in 5%, 18% and 4% cases, respectively [91]. COVID-19 patients who develop frequent PVCs (> 120 per 24 h) should be monitored carefully by assessing pro-BNP (Brain Natriuretic peptide) and hs-cTn (High Sensitivity Cardiac Troponin) which might potentially reveal the occurrence of overt myocardial injury [91]. Any consistent elevation of these markers can herald the onset of PVC induced cardiomyopathy manifesting as progressive heart failure characterized by LV dilation and dysfunction in the absence of major cardiac abnormality and complete reversal post successful catheter ablation of PVCs [92].

Recently, a patient with protracted COVID-19 infection that presented with chest pain, palpitations and shortness of breath was found to have PVC bigeminy and elimination of these with catheter ablation resulted in successful clinical outcomes [93]. In a prospective analysis performed in Shahid Mostafa Khomeini Hospital of Ilam in Iran from March to August, 2020, it was revealed that A-Fib and PVC (20%) are the most common arrhythmias followed by VT (4.44%) and VF (2.22%) [94]. In a worldwide retrospective analysis of hospitalized COVID-19 patients, atrial fibrillation, NSVT, VT and VF occurred in 62%, 9.2%, 8.2% and 4% of these cases, respectively [95]. In a prospective observational study performed in the hospitalized COVID-19 patients from March 15 to April 30, 2020, subjected to telemetry, the incidence of ventricular arrhythmias include PVC (28.7%), NSVT (15.4%), VT (1.4%) and VF (0.7%), respectively [96]. Another interesting finding in this study is that new-onset atrial fibrillation and serious ventricular arrhythmias only occurred in patients with elevated troponin levels [96]. Since COVID-19 virus is notorious to inflict myocarditis, concomitant presence of benign (PVC, VT and NSVT) and malignant (VF) ventricular arrhythmias is bound to be expected [96, 97]. In a recent case report, a 68-year-old Caucasian women hospitalized for COVID-19 infection developed electrical storm due to sustained and non-sustained VT, which were not controlled by implantable cardioverter defibrillator (ICD) shock therapy [98]. Eventually, she underwent substrate-based VT catheter ablation resulting in complete resolution of arrhythmias [98]. The authors argue that a combination of factors ranging from myocardial involvement, cytokine storm, hypoxia, intracellular calcium overload to early after-depolarization might be responsible for manifestation of ventricular arrhythmias during the clinical course of COVID-19 patients [98].

### Is there a link between the vaccination and COVID-19-induced A-fib?

New-onset A-fib is most commonly associated with Pfizer followed by Moderna and Johnson and Johnson vaccines [99]. According to Vaccine Adverse Reporting System (VAERS), after administration of 532 million doses of vaccine, the number of total and new-onset A-fib cases reported is 2611 and 315, respectively [99]. The average incidence of A-fib cases is approximately 5 per 1 million doses vaccine administered [99]. Another meta-analysis study indicated that the incidence of A-fib post COVID-19 vaccination is around 1–76 per 10,000 vaccine doses administered [100]. It is being speculated that vaccine administration leads to local and systemic changes including ion channel aberrations, calcium dys-homeostasis and atrial remodeling, thereby priming the myocardial cells for developing A-fib [101]. Moreover, it is not possible to rule out the development of autoimmunity and subsequent deposition of immune complexes in atrial myocardium in the pathogenesis of atrial fibrillation post vaccination in the COVID-19 patients. Furthermore, the stress, anxiety and fear associated with vaccination were indirectly linked to the development of A-fib in the COVID-19 patients [102]. Mechanistically, these negative emotions tend to destabilize the autonomic nervous system by energizing the sympathetic arm while deactivating the parasympathetic component, thereby kick-starting arrhythmogenic pathways that are pivotal in provoking atrial fibrillation even in patients without structural heart disease [102]. On the contrary, vaccination in the hospitalized patients with COVID-19 reduced risk of developing A-fib by 12% (adjusted HR = 0.88, 95%CI 0.79–0.93) [103].

### Future directions for research

**Fig. 5:**
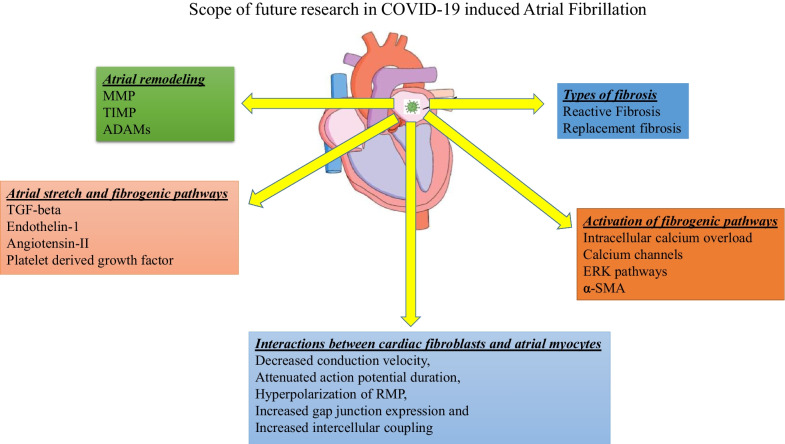
Scope of future research in COVID-19 induced research

Deposition of fibrous tissue, scar formation and generation of atrial substrate in the atrial myocytes are hypothesized to be some of the most critical driving mechanisms for initiating and sustaining A-Fib. Fundamentally, two main types of fibrosis namely reactive fibrosis and replacement fibrosis can potentially occur in atrial chambers in pathological states [104] (Fig. 5). Reactive fibrosis is process where collagen deposition occurs in the periphery of cardiomyocytes, whereas replacement fibrosis occurs when collagen accumulation tend to replace the dying cardiomyocytes within the myocardium [104]. Anatomically, with fibrous tissue interspersing between the myocytes there might be conduction disturbances more commonly associated with replacement fibrosis than reactive fibrosis [104]. It has been speculated that pathological disease processes occurring in atrial chambers tend to incite both these types of fibrosis in varying proportions and their relative ratio is very crucial in determining whether they pave the way toward development of atrial arrhythmias [104]. Therefore, it would be prudent to investigate the relative percentages of reactive and replacement fibrosis in COVID-19-infected cardiomyocytes in atrial chambers. Identifying the threshold of atrial fibrosis that is necessary for triggering of atrial fibrillation/flutter with COVID-19 infections would be beneficial. This will eventually shed light on the underlying mechanisms that predispose to development of atrial substrate, a key molecular finding that incites atrial fibrillation. Furthermore, it would be worthwhile to characterize the influence of COVID-19-induced atrial fibrosis on atrial myocyte electrophysiological properties such as conduction, ectopic generation, reentry mechanisms, refractory period and action potential duration [105] (Fig. 5).

Previous studies suggest that calcium entry in atrial fibroblast is very important for their survival and activation of fibrogenic pathways [104]. The main incentive for calcium gaining access into atrial fibroblasts is their resting membrane potential (RMP), which is primarily regulated by inward rectifier potassium current (*I*_k1_) [106]. Pathological process such as heart failure tends to hyperpolarize the RMP of atrial fibroblasts by activating *I*_k1_ currents and thus provides a potent stimulus for enhanced calcium entry [106]. Extracellular calcium enters into atrial fibroblasts through three important channels, namely store-operated channels (SOC), receptor-operated channels (ROC) and transient receptor potential (TRP) channels, and causes extracellular signal-related kinase (ERK) pathway mediated activation leading to stimulation of fibrogenic pathways as well as fibroblast survival [104, 107, 108]. Future research studies should be focused on investigating the effects of COVID-19 virus on RMP, calcium channels and ERK pathways in atrial fibroblasts (Fig. 5). This might provide indirect information regarding the downstream signaling mechanisms that underlie enhanced fibrogenesis and atrial substrate formation in atrial myocardium for development of atrial fibrillation.

In vitro study performed to assess the interactions between fibroblasts isolated from infarcted hearts and neonatal myocytes, there is decreased conduction velocity, attenuated action potential duration, hyperpolarization of RMP, increased gap junction expression and increased intercellular coupling between myocytes and fibroblasts [109] (Fig. 5). Accordingly, enhanced interactions between cardiomyocytes and fibroblasts are shown to enhance ectopic impulse generation and facilitate generation of re-entrant arrhythmias [104, 110, 111]. In contrast to these findings, increasing the intercellular interactions between cardiac fibroblasts and cardiomyocytes by increasing the expression of connexin-43 reduced the risk of developing ventricular tachycardia post myocardial infarction [112]. Therefore, we can speculate COVID-19-induced myocardial injury might alter intercellular interactions between upregulated fibroblasts and atrial myocytes. Characterizing the downstream effects of these intercellular interactions between them and assessing their end targets would unravel the underlying pathogenic mechanisms for development of atrial arrhythmias in COVID-19-induced myocardium (Fig. 5).

Mechanical stretch of cardiomyocytes stimulates the secretion of pro-fibrotic signals such as angiotensin-II, transforming growth factor (TGF)-1β, endothelin-1 (ET-1) and platelet-derived growth factor (PDGF) [105] (Fig. 5). Pro-fibrotic signals act upon neighboring fibroblasts in a paracrine fashion to enhance the synthesis of collagen and other extracellular matrix (ECM) proteins and thus causing atrial fibrosis, and atrial fibrillation [105]. Clinical evidence indicating that angiotensin-II is involved in facilitating cardiac fibrosis comes from the fact that losartan (ACE inhibitor) is routinely prescribed in heart failure for suppress cardiac fibrosis in humans [113]. Furthermore, ACE inhibitors and AT-1 receptor antagonists were marginally successful in attenuating atrial fibrogenesis and suppressing A-Fib in animal models [105]. TGF-1β is hypothesized to act via TGF-1β-activated kinase (TAK-1) for causing cardiac fibroblast induced ECM and collagen production leading to cardiac fibrosis post cardiac injury [113]. PDGF is known to act through enhancing cardiac fibroblast proliferation as well as TGF-1β expression, both of which are conducive for progression of cardiac fibrosis post cardiac injury. Efforts made to neutralize the downstream signaling events of PDGF through application of PDGF antibody had yielded successful outcomes in downgrading atrial fibrosis in heart failure model [114]. ET-1 is shown to enhance the collagen production by cardiac fibroblasts and functions as a secondary messenger for Angiotensin-II and TGF-1β for mediating cardiac fibrosis [113]. Blockade of ET-1 signaling might be beneficial in halting signaling pathways that mediate atrial fibrosis and can be a potential therapeutic strategy for preventing atrial fibrillation. In a single-center observational study of COVID-19 patients from February 10 to March 13, 2020, echocardiographic follow-up revealed that 57.9% of these patients had increased left atrial (LA) size [115]. This increase in left atrial size would cause stretch in the atrial myocytes and would eventually make them to secrete pro-fibrotic signals leading to interactions with adjacent fibroblasts. Therefore, it would be prudent to investigate whether these pro-fibrotic signals would be upregulated with COVID-19 infection of atrial myocytes and to investigate the interactions of these pro-fibrotic signals with adjacent fibroblasts.

The relationship between atrial fibrosis and atrial arrhythmogenicity is complex, but few previous studies tried to partially explain it by intracellular calcium overload, oxidative stress, α-SMA (Alpha- Smooth Muscle Actin) over expression and exaggerated myocyte–fibroblast coupling leading to electrical remodeling, enhanced arrhythmogenesis, ectopic impulse generation and abnormal impulse conduction [116–119] (Fig. 5). So, studies focused on assessing whether COVID-19 infection of cardiomyocytes generates cellular abnormalities would be beneficial so that appropriate therapeutic interventions can be designed for halting the generation of atrial arrhythmias.

Inflammation and oxidative stress has been documented to a play a significant role in regulating the role of enzymes and tissue factors that regulate the synthesis and degradation of collagen matrix deposition in the myocardial tissues [119]. Previous studies indicate that pro-inflammatory cytokines (TNF-alpha and IL-1 beta) modify the expression of tissue inhibitors of matrix metalloproteinases (TIMP) and matrix metalloproteinases (MMPs) in such a way that there is decreased collagen production and increased collagen degradation [120, 121] (Fig. 5). Oxidative stress with production of ROS has been implicated in promoting fibroblast proliferation, activation of intermediate signaling molecules (ERK, P-38 and JNK), and upregulation of nuclear transcription of pro-fibrotic genes. This might result in increased extracellular matrix (ECM) deposition and collagen in myocardial tissues [122, 123]. Oxidative stress and inflammatory cytokines are implicated in the disruption of MMP regulation in the cardiomyocytes and cardiac fibroblasts, thereby increasing the risk of remodeling and fibrosis in atrial tissues [124–126]. For example, IL-1 beta and TNF-alpha are some of the important regulators of MMP-9 transcription and translation, which is previously incriminated in atrial remodeling and fibrosis [127]. Analysis of atrial tissues revealed increased MMP-9 mRNA and protein expression and down-regulated expression of TIMP-1 was associated with increased atrial remodeling and increased propensity toward developing A-fib [128, 129]. These above findings highlight the importance of MMPs and TIMPs in physiological regulation of ECM deposition and collagen synthesis.

Since COVID-19 infection of atrial cardiomyocytes is associated with cytokine storm [6] and oxidative stress [119], it is quite possible that there is underlying imbalance in synthesis of MMPs and TIMPs in the atrial tissues, thereby causing atrial remodeling and fibrosis. Therefore, assessing the expression of MMPs and TIMPs in atrial myocytes of patients developing COVID-19-induced A-fib would shed some light by providing valuable information regarding pathogenesis of atrial fibrosis.

ADAMs (*A D*isintegrin *A*nd *M*etalloproteinase) are membrane bound glycoproteins that are expressed in myocardial tissues (Fig. 5). Their main function is to regulate the intercellular and cell–matrix interactions in the atrial and ventricular myocardium. Their accessory functions can range from proteolysis, adhesion, cleavage and fusion. In physiological conditions, ADAMs bind to integrins for modulating cell motility, adhesion and migration of fibroblasts [130]. Alteration of intercellular and cell–matrix interactions was previously demonstrated in physiological and pathological conditions of heart [131, 132]. Analysis of atrial tissues from 30 patients undergoing open heart surgery revealed that there is increased expression of ADAM10 and ADAM15 as well as increased ADAM /integrin ratios in the fibrillating atrial tissues compared to patients with no history of AF [133]. These researchers argue that increased ADAM expression induced structural and transport changes might be accountable for atrial dilation seen in atrial fibrillation [133]. Specifically, they hypothesize that modification of expression and interactions of ADAM 10 and 15 with integrins β_1_ and β_3_ might lay the foundations for structural remolding and subsequent dilation in fibrillating atria [130]. These findings make us to contemplate that COVID-19 infection of atrial myocytes might induce changes in ADAMs resulting in altered atrial myocyte–cell matrix interactions leading to atrial dilation and atrial fibrillation. Investigating this hypothesis would enlighten us regarding the influence of ADAMs in pathogenesis of COVID-19-induced A-fib.

## Conclusions

A-fib usually occurs acutely in COVID-19 patients admitted to ICU, particularly in those with high-risk factors. On the contrary, some patients presented with atrial fibrillation few months later after complete recovery from COVID-19 infections. These long haulers are known to harbor silent COVID-19 infections in the systemic tissues including myocardium. Clinicians need to be vigilant of this arrhythmia as it can be associated with many life-threatening complications. Prompt management and close monitoring are necessary as it has high chances of recurrence. COVID-19-induced pathological derangements in the myocardium and systemic circulation in combination are accountable for instigation and persistence of this cardiac arrhythmia. This review summarizes the pathophysiology of COVID-19-induced A-fib along with its clinical presentation, diagnosis, management and complications. Understanding the interplay of various risk factors both extra-cardiac and intra-cardiac as well as cellular downstream signaling events is very crucial. Since this is a relatively clinical entity, there is a lot of scope in research to understand its pathophysiology. We summarized the various areas where basic science and clinical research studies need to be concentrated in the near future. Execution of these research studies can generate very useful preliminary data that can help us to comprehend necessary downstream signaling events that happen at the cellular level for initiation and perpetuation of atrial fibrillation in COVID-19 patients. Moreover, the molecular targets that are divulged by these research studies can be exploited for crafting novel diagnostic and therapeutic interventions for reducing morbidity and mortality of this fatal cardiac arrhythmia.

## Supplementary Information

**Additional file 1**. Drug-drug interactions in the management of Atrial fibrillation encountered in COVID-19 patients.

## Data Availability

No data are presented and analyzed in this manuscript.

## Abbreviations

ICU: Intensive care unit
A-fib: Atrial fibrillation
MACE: Major adverse cardiovascular events
ECG: Electrocardiogram
NSVT: Non-sustained ventricular tachycardia
ACE2: Angiotensin-converting enzyme 2
RAC3: Ras-related C3 botulinum toxin substrate3
STAT3: Signal transducer and activator of transcription-3
CX40: Connexins40
RyR: Ryanodine receptors
SERCA: Sarco-endoplasmic reticulum calcium ATPase
PLB: Phospholamban
*I*_CaL_: Inward depolarizing calcium current
*I*_Na_: Inward depolarizing sodium current
*I*_Ks_: Slow delayed potassium current
*I*_Kr_: Delayed rectifier potassium current
*I*_to_: Transient outward potassium current
Th1&2 lymphocytes: Thymic lymphocytes 1&2
IL-6: Interleukin-1
IL-1: Interleukin-1
CRP: C-reactive protein
HERG K+ channel: Human ether-a-go-go-related gene K+ channel
EAD: Early after depolarization
DAD: Delayed after depolarization
*I*_k1_: Inward rectifier potassium current
MV: Mechanical ventilation
CCBs: Calcium channel blockers
CHF: Congestive Cardiac Failure
AFEQT: Atrial fibrillation effect on quality of life
TF: Tissue factor
TIA: Transient ischemic attack
ASSERT study: Asymptomatic atrial fibrillation and stroke evaluation in pacemaker patients and the atrial fibrillation reduction atrial pacing trial
VTE: Venous thromboembolism
PE: Pulmonary embolism
LA: Left atrium
LAA: Left atrial appendage
RAA: Right atrial appendage
AV node: Atrioventricular node
WPW: Wolf Parkinson White Syndrome
PVC: Premature ventricular contractions
VT: Ventricular tachycardia
VF: Ventricular fibrillation
ICD: Implantable cardioverter defibrillator
RMP: Resting membrane potential
SOC: Store-operated calcium channels
TRP: Transient receptor potential
ERK: Extracellular signal-related kinase
ET-1: Endothelin-1
PDGF: Platelet-derived growth factor
ECM: Extracellular matrix
TGF-1β: Transforming growth factor-1 beta
TAK1: TGF-1β-activated kinase
SMA: Smooth muscle actin
TIMP: Tissue inhibitors of matrix metalloproteinases
MMP: Matrix metalloproteinases
JNK: C-Jun N-terminal kinase
ADAMs: *A D*Isintegrin *A*nd *M*etalloproteinase
